# Unexpected seronegative response in relapsed PLA2R-associated membranous nephropathy

**DOI:** 10.1093/ckj/sfae189

**Published:** 2024-06-21

**Authors:** Wing Yin Leung, Henry H L Wu, Beena Nair, Alexander Woywodt, Arvind Ponnusamy

**Affiliations:** Department of Renal Medicine, Lancashire Teaching Hospitals NHS Foundation Trust, Preston, UK; Renal Research, Kolling Institute of Medical Research, Royal North Shore Hospital & The University of Sydney, Sydney, NSW, Australia; Department of Pathology, Lancashire Teaching Hospitals NHS Foundation Trust, Preston, UK; Department of Renal Medicine, Lancashire Teaching Hospitals NHS Foundation Trust, Preston, UK; Faculty of Biology, Medicine & Health, The University of Manchester, Manchester, UK; Department of Renal Medicine, Lancashire Teaching Hospitals NHS Foundation Trust, Preston, UK; Faculty of Biology, Medicine & Health, The University of Manchester, Manchester, UK

To the Editor,

The M-type phospholipase A2 receptor (PLA2R), a transmembrane receptor on glomerular podocytes, was identified as the major antigenic target in idiopathic membranous nephropathy (IMN) in 2009 [1]. Since then, circulating autoantibodies of PLA2R (PLA2RAb) have emerged as a diagnostic marker in IMN, and as a tool to monitor disease activity and response to treatment. Depletion of PLA2RAb usually indicates immunological remission, and relapse is likely when titres rise again [2, 3]. We present the unusual case of a 63-year-old woman who was previously treated with rituximab for PLA2R-positive membranous nephropathy (MN), who first relapsed with positive PLA2R and then sustained a second relapse without a rise in PLA2RAb titre.

The patient first presented in July 2012 with nephrotic syndrome, where urine protein–creatinine ratio (uPCR) was 1333 mg/mmol and serum albumin was 24 g/L. With a positive PLA2RAb [indirect immunofluorescence test (IIFT) 100 titre units], she was diagnosed with PLA2R-positive MN which was also confirmed by kidney biopsy. Kidney biopsy did not suggest chronicity of disease. The patient received 8 months of treatment with the Ponticelli regimen, where partial remission of disease was achieved. She was diagnosed with polymyositis in August 2013, and was commenced on prednisolone and azathioprine.

The patient had an initial relapse episode in mid-2018 with uPCR 697 mg/mmol, serum albumin 34 g/L and PLA2RAb positivity [IIFT 100 titre units, enzyme-linked immunosorbent assay (ELISA) 51 RU/mL]. She subsequently received two doses of 1 g rituximab and serological remission was achieved. Her kidney function remained stable with estimated glomerular filtration rate (eGFR) 40 mL/min/1.73 m^2^.

**Figure 1:**
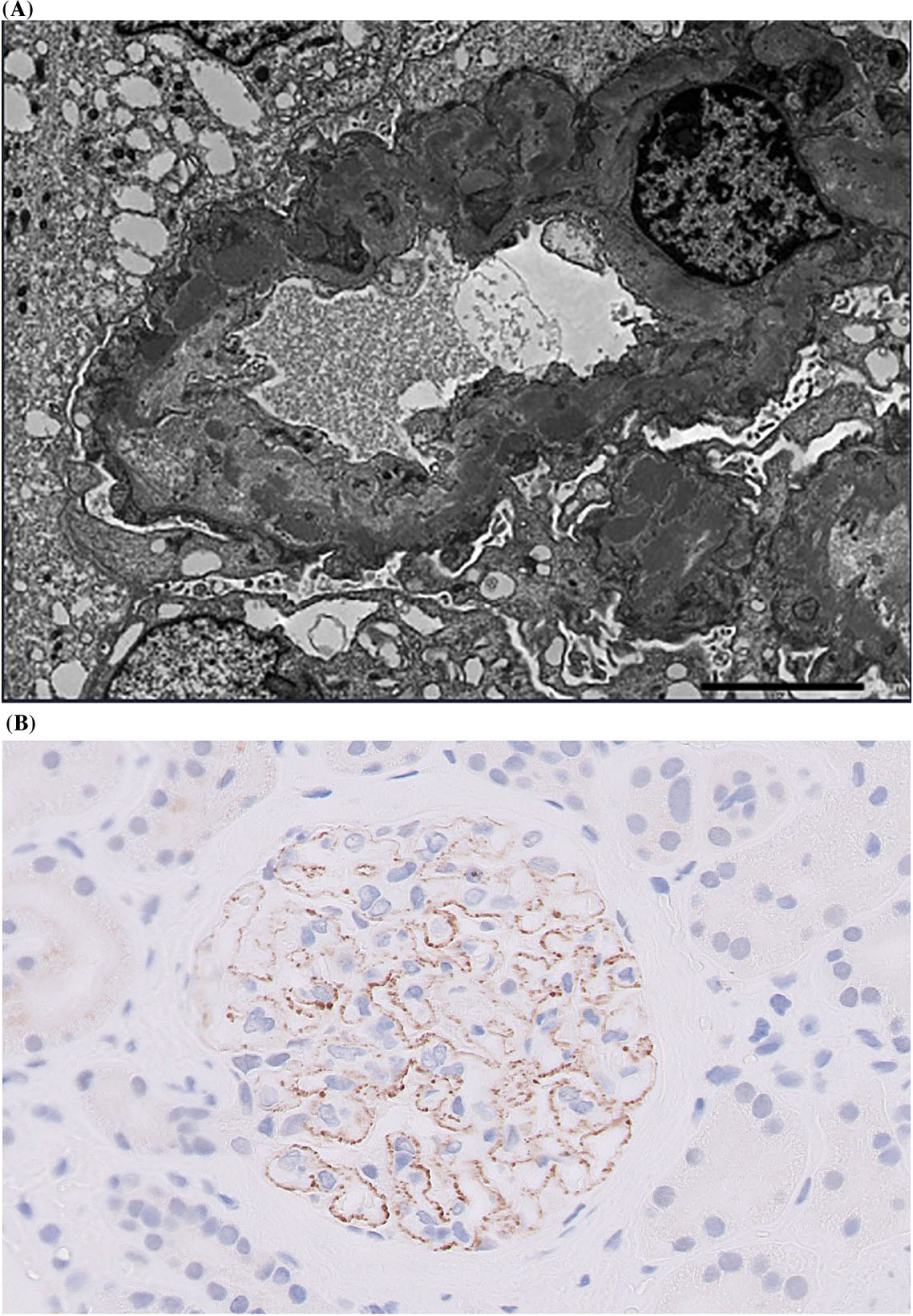
Kidney biopsy findings during the second relapse episode of PLA2R-associated membranous nephropathy. (**A**) Electron microscopy of kidney biopsy tissue displaying subepithelial electron-dense deposits along glomerular basement membrane; (**B**) Positive immunostaining on kidney biopsy suggestive of PLA2R-positive membranous glomerulonephritis (at ×40 magnification).

The second relapse episode occurred in September 2022, when proteinuria levels returned to a nephrotic range (uPCR 650 mg/mmol and serum albumin 38 g/L). It was surprising, however, that her serum PLA2RAb appeared negative in both assay immunofluorescence and ELISA on this occasion. Despite the optimization of her supportive treatment regimen including anti-proteinuric therapy and tight blood pressure control, the patient's proteinuria remained in the nephrotic range following 9 months of treatment. There was slight decline in kidney function with eGFR 32 mL/min/1.73 m^2^, albeit with preserved serum albumin. Malignancy was ruled out. A repeat kidney biopsy demonstrated features suggestive of MN—electron microscopy displayed new subepithelial deposits (Fig. 1A), but no features of scarring that would have accounted for proteinuria; immunostaining of kidney biopsy tissue confirmed PLA2R-positive membranous glomerulonephritis (Fig. 1B). In the second relapse episode, our patient responded well to rituximab (a cumulative dose of 2 g was administered, which was slowly infused with split dosages required due to a previous mild reaction to rituximab) and partial remission in her proteinuria was achieved. Subsequent serum PLA2RAb measurements have remained negative to date.

Current evidence suggests PLA2RAb positivity is present in approximately 70% of IMN cases [3, 4]. As immunological changes conventionally precede clinical course of disease, PLA2RAb positivity is frequently observed prior to onset of clinical manifestations in MN [3]. Serial monitoring of PLA2RAb has been deemed to be a reliable tool during follow-up to determine the status of recurrent disease. At present, there remains no published evidence providing a clear explanation relating to cases of biopsy-positive, but seronegative PLA2R-associated MN. B-cell-depletion therapies such as rituximab, now commonly applied as an attractive first-line treatment option in MN due to its favourable efficacy and safety profile, has been previously established to reduce antibody response [5]. We postulate here that our case patient's use of rituximab may have ‘masked’ their PLA2RAb response. Our case suggests an important caveat in the role of routine serial PLA2RAb monitoring and emphasizes the role of repeat kidney biopsy in relapsed MN [2]. Further studies should delineate clinical characteristics of similar patients and report outcomes to improve our understanding of this unusual scenario.
